# Antinociceptive activity of doliroside B 

**DOI:** 10.1080/13880209.2022.2163407

**Published:** 2023-01-10

**Authors:** Xishan Bai, Yanhong Li, Yuxiao Li, Min Li, Ming Luo, Kai Tian, Mengyuan Jiang, Yong Xiong, Ya Lu, Yukui Li, Haibo Yu, Xiangzhong Huang

**Affiliations:** a Key Laboratory of Chemistry in Ethnic Medicinal Resources, State Ethnic Affairs Commission & Ministry of Education, School of Ethnic Medicine, Yunnan Minzu University, Kunming, China; b State Key Laboratory of Bioactive Substance and Function of Natural Medicines, Institute of Materia Medica, Chinese Academy of Medical Sciences and Peking Union Medical College, Beijing, China

**Keywords:** Analgesic mechanism, COX-1, GABA_A1_ receptor

## Abstract

**Context:**

*Dolichos trilobus* Linn (Leguminosae) is often used in Yi ethnic medicine to treat pain, fracture, and rheumatism.

**Objective:**

To explore the therapeutic potential of doliroside B (DB) from *D. trilobus* and its disodium salt (DBDS) and the underlying mechanism in pain.

**Materials and methods:**

In the writhing test, Kunming mice were orally treated with DB and DBDS at doses of 0.31, 0.62, 1.25, 2.5, and 5 mg/kg. Vehicle, morphine, indomethacin, and acetylsalicylic acid were used as negative and positive control on the nociception-induced models, respectively. In the hot plate test, mice were orally treated with DB and DBDS at doses of 2.5, 5, 10, and 20 mg/kg. In the formalin test, mice were orally treated with DB and DBDS at doses of 2.5, 5, 10, and 20 mg/kg. In the meanwhile, lipopolysaccharide-induced inflammatory model in RAW264.7 macrophages was adopted to study the mechanism of pain alleviation for DBDS.

**Results:**

DBDS (5 mg/kg) inhibited the writhing number by 80.2%, which exhibited the highest antinociceptive activity in pain models. DBDS could selectively inhibite the activity of COX-1. Meanwhile, it also reduced the production of NO, iNOS, and IL-6 by 55.8%, 69.0%, and 49.9% inhibition, respectively. It was found that DBDS also positively modulated the function of GABA_A1_ receptor.

**Discussion and conclusions:**

DBDS displayed antinociceptive activity by acting on both the peripheral and central nervous systems, which may act on multitargets. Further work is warranted for developing DBDS into a potential drug for the treatment of pain.

## Introduction

Pain causes a negative consequence on health status and influences the quality of life (Williams and Craig 2016). As pain is a complex medical concern, multiple pathways involved in the process from the transduction of noxious stimuli to cognitive and emotional processing in the brain (Ossipov et al. 2014). To date, many drugs are used in clinic management of pain symptoms, such as non-steroidal anti-inflammatory drugs (NSAIDs), opioid drugs, and anti-depressive drugs. Among these drugs, opioid analgesics are the most effective pharmacological agents for moderate to severe pain. Unfortunately, their therapeutic benefit is often limited by analgesic tolerance and hyperalgesia (Benyamin et al. 2008). Unwanted side effects also occur very often. In this sense, natural products could serve as a better choice for pain management drug discovery.

Traditional Yi medicine, as an ancient and still vital holistic system, has made great contributions to the health of the Yi people distributed mainly over Yunnan, Sichuan and Guizhou provinces, China, receiving much wider recognition for its own unique theories of human health and therapeutics (Xu et al. 2015; Gao and Yang 2018). Due to the special climate in the zone Yi people live, there is a high incidence of pain-related diseases, such as arthritis and rheumatism. Hence, Yi doctors have accumulated a wealth of knowledge and experience in the treatment of these diseases (Bi et al. 2018). As a representative of Yunnan Yi medicine, the radix and rhizoma of *Dolichos trilobus* Linn (Leguminosae) have been widely used to treat pain, fracture, and rheumatism for many years (Lan et al. 2015). It has been previously reported that the saponins of *D. trilobus* exhibited a few bioactivities such as anti-inflammation (Chen et al. 2013; Wei et al. 2014), analgesic (Huang et al. 1982b), antitumor (Huang et al. 1982a), and suppression of amyloid β-protein 42 fibrillogenesis (Li et al. 2017). However, there is still limited information available for its analgesic constituents and mechanism. Therefore, identifying effective bioactive constituents with antinociceptive effects from it is very possible. Interestingly, doliroside B (DB, a triterpenoid saponin) is an important component in the roots of this plant with high content in the range of 0.26 to 10.49% (Ma et al. 2015), around 86.6% of the total saponins of *D. trilobus* (Xu et al. 2015). The antinociceptive activity of triterpenoid saponins has been widely reported (Speroni et al. 2007; Arrau et al. 2011; Cheng et al. 2016; Mao et al. 2016; Chen et al. 2018; Sun et al. 2019). Therefore, we speculated that compound DB might be the main analgesic constituent of *D. trilobus* for the treatment of pain. In order to test the effect of *D. trilobus* on pain alleviation, we examined the antinociceptive activity of *D. trilobus* extract, compound DB isolated from the extract and its disodium salt (DBDS) in the present research. Meanwhile, the probable antinociceptive mechanisms involved in DBDS activity were also explored.

## Materials and methods

### Chemicals and reagents

Acetic acid was purchased from Guangdong Guanghua Sci-Tech Co., Ltd. Acetylsalicylic acid (ASA), indomethacin (IND), and morphine (MOP) were purchased from National Institutes for Food and Drug. Naloxone hydrochloride (NAL) and formalin were purchased from J&K Scientific Ltd. Materials for cell culture were purchased from Yunnan Chien Technology Co., Ltd. ELISA Kit were purchased from Bio-Techne China Co., Ltd. Griess reagent and NG-Monomethyl-l-arginine acetate (L-NMMA) were purchased from Beyotime Biotechnology Co., Ltd.

### Plant material

During September 2019, the roots of *D. trilobus* were collected at Chuxiong City, Yunnan Province, China. It was authenticated by Prof. Qing-Song Yang at the Yunnan Minzu University. The voucher specimen (No. 20190928) was deposited at the Key Laboratory of Chemistry in Ethnic Medicinal Resources, State Ethnic Affairs Commission & Ministry of Education, School of Ethnic Medicine, Yunnan Minzu University.

### Extraction, isolation and preparation of DBDS

According to previously reported procedures by our group (Luo et al. 2021), we prepared the ethanol extract. The air-dried and finely powdered roots (10 kg) of *D. trilobus* were macerated in 95% ethanol (3 × 60 L) for 48 h at room temperature. After filtering and evaporating the extract to remove ethanol, the remaining dark brown residue was retained (1.15 kg). DB (33.4 g, yield: 2.9%, m.p. 252–253 °C) was isolated from the *D. trilobus* extract by recrystallization (methanol). DB (20 g) was dissolved in methanol at room temperature followed by addition of 2 equiv. of NaOH for the production of DBDS. After NaOH was dissolved completely, the solution was concentrated under reduced pressure to provide the desired product DBDS (20.1 g, yield: 94.3%). The ^1^H and ^13^C NMR spectra of DB and DBDS were shown in Supplementary material.

### Animals

Female Kunming mice (20 ± 2 g) from Chengdu Dossy Experiment Animal Co., Ltd. (Sichuan, China) were used for antinociceptive experiments. These animals were kept under a constant temperature (23 ± 1 °C), humidity (40–70%), airflow speed (0.1–0.2 m^3^/s) and noise (≤ 60 db), and maintained under a 12 h light-dark cycle. After the experiment is completed, mice were euthanized humanely. All the animal experiments were approved by the Institutional Animal Care and Use Committee of Yunnan Minzu University (permit no. 20200608).

### Acetic acid-induced writhing test

Acetic acid-induced writhing test (Koster 1959) was employed to evaluate the analgesic effect of *D. trilobus* extract, DB and DBDS. Briefly, healthy female mice which are more susceptible to the development of pain (Lu and Yan 1996) were randomly divided into seven groups (*n* = 10), each group was pretreated with vehicle (distilled water), MOP (1 mg/kg), IND (10 mg/kg), ASA (100 mg/kg), and *D. trilobus* extract (200, 400, and 800 mg/kg) by oral or intraperitoneal administration, respectively. After intragastric (i.g.) or intraperitoneal administration of 30 min, mice were intraperitoneally (i.p.) injected with 0.6% acetic acid (0.2 mL). Following acetic acid injection, the number of writhing induced by acetic acid were counted cumulatively for 15 min. Employing the above mentioned procedures, mice were randomly divided into six groups (*n* = 10), we evaluated the antinociceptive effect of DB, DBDS, IND, ASA, and MOP by treating mice with vehicle and different dose of test compounds, respectively. Among the tests in the absence and presence of NAL (5 mg/kg), i.p. injection of NAL was performed 40 min before acetic acid injection. The inhibition was calculated using the formula: inhibition (%) = (average of control group – average of test group)/average of control group × 100%.

### Eddy’s hot plate test

This was performed according to a previously reported procedure (Eddy and Leimbach 1953). The pain threshold was defined as the duration from the beginning of placing mice on the plate to the mice jumping, licking, or shaking its paws. Because the scrotum of male mice is saggy, which can be stimulated by hot plate, healthy female mice with pain threshold less than 20 s were used for hot plate test. Mice were randomly divided into twelve groups (*n* = 10), each group was pretreated with vehicle (distilled water), MOP (1 mg/kg, positive control), IND (10 mg/kg, positive control), ASA (100 mg/kg, positive control), DB (2.5, 5, 10, and 20 mg/kg), and DBDS (2.5, 5, 10, and 20 mg/kg) by oral or intraperitoneal administration, respectively. After administration of the test compounds, the pain threshold was recorded at 0, 30, 60, 90, and 120 min. Among the tests in the absence and presence of NAL (5 mg/kg), i.p. injection of NAL was performed 10 min before administration of testing compounds. After administration of the test compounds, the pain threshold was recorded at 0, 30, 60, 90, and 120 min.

### Formalin test

The test was performed as described by Ayumi et al. (2020). Female mice were randomly divided into twelve groups (*n* = 6), each group was pretreated with vehicle (distilled water), MOP (1 mg/kg, positive control), IND (10 mg/kg, positive control), ASA (100 mg/kg, positive control), DB (2.5, 5, 10, and 20 mg/kg), and DBDS (2.5, 5, 10, and 20 mg/kg) by oral or intraperitoneal administration, respectively, 1 h before the formalin injection. 2.5% Formalin (20 μL) was injected in the plantar surface of the right hind paw. The indicator of pain was evaluated as the time spent licking paw. It was recorded from 0 to 5 min (early phase) and from 15 to 30 min (late phase) after the injection of formalin. Among the tests in the absence and presence of NAL, i.p. injection of NAL (5 mg/kg) was performed 10 min before administration of testing compounds.

### Cell culture

The RAW264.7 cell line (purchased from Kunming Institute of Zoology. Chinese Academy of Sciences) was cultured in DMEM (Dulbecco’s modified Eagle medium) supplemented with 10% FCS (fetal calf serum) under a humidified atmosphere (5% CO_2_, 37 °C).

To study the antinociceptive mechanism of the test compound, the following cells were used, including GABA_A_ α1β2γ2-T-REx™-CHO, Nav1.7-HEK-FlpIn, Nav1.2-CHO-FlpIn, Nav1.4-CHO-K1, Nav1.5-HEK293, and TRPV1 (transient receptor potential cation channel subfamily V member 1)-HEK293 cells. All the ion channel-expressed cell lines were made in Haibo Yu’s lab. Among the parental cells, T-REx™-CHO, CHO-FlpIn, and HEK-FlpIn were obtained from Thermo Scientific; CHO-K1 and HEK293 cells were obtained from ATCC. CHO-derived cells were cultured in DMEM and nutrient F-12 mixture, and HEK293-derived cells were cultured in DMEM supplemented with 10% FCS. For GABA (γ-aminobutyric acid) cells, the following antibiotics were added, blasticidin (10 μg/mL), hygromycin (300 μg/mL), zeocin (100 μg/mL), and puromycin (1 μg/mL). For Nav1.2-CHO-FlpIn and Nav1.7-HEK-FlpIn, hygromycin (300 μg/mL) was added. For Nav1.4-CHO-K1, Nav1.5-HEK293, and TRPV1-HEK293 cells, geneticin (500 μg/mL) was added. Stable cell lines with highly-expressed channels or receptors are cultured in the designated medium, and cultured in a humidified incubator (5% CO_2_, 37 °C). Cells were passaged every 3–4 days.

### Fluorescence-based assay

On the day before screening, the cells were seeded in 384-well plates (CHO-derived cells, seeding density at 15,000 cells/well; HEK-293-derived cells, seeding density at 18,000 cells/well) and cultured overnight. The assay protocol was adapted from the manufacturer’s recommended protocol. Briefly, the medium was removed; cells were loaded with the designated dye, 20 μL/well, and incubated for 30 min [FMP (Fluorescence-based Membrane Potential) dye] and 60 min (Calcium 6 dye) at room temperature in the dark. After dye loading, the test compounds were added into the cell plate and incubated for 20 min. Cell plates were loaded to FLIPR machine (Molecular Devices, USA). After establishing fluorescence baseline by 1 Hz scanning for 10 s, the channels were activated by the addition of agonist or test compounds (5 μL, 5-fold), and fluorescence measurement was continued at 1 Hz for another 120 s. EC_50_ (half effective concentration) values were determined from dose-response curves for 8 distinct concentrations in quadruplicates. The concentration-response curves were fitted by Hill equation, according to the standard procedure.

### Cell viability assay

The MTT (3-(4,5-dimethylthiazol-2-yl)-2,5-diphenyltetrazolium bromide) colorimetric method was used to determine cytotoxicity of DBDS. The logarithmic phase cells were seeded in 96-well plates with 1 × 10^5^ cells/well (volume of the well is 100 μL). Under a humidified atmosphere (5% CO_2_, 37 °C), the cells were cultured for 24 h following treatment with vehicle and DBDS (12.5, 25, 50, 100, and 200 µmol/L). After 24 h, 20 μL of MTT solution was added to each well and kept under the same environment for 4 h again. Subsequently, 150 μL of DMSO was added to each well after removing the culture supernatant, vibrated and kept under a darkroom at room temperature for 15 min. The optical density (OD) of the solution was measured with a microplate reader at 490 nm. The survival rate was calculated using the formula, survival rate (%) = OD of the sample-treated group/OD of the control group × 100%.

### NO inhibitory assay

A modified method, according to previous report by Banskota et al. (2003), was employed for the evaluation of inhibitory effect of test compounds on nitric oxide (NO) production in RAW264.7 cells. Briefly, the logarithmic phase cells were seeded in 96-well plates with 1 × 10^5^ cells/well (volume of the well is 100 μL). Under a humidified atmosphere (5% CO_2_, 37 °C), the cells were cultured for 24 h. Subsequently, 50 μL of lipopolysaccharide (LPS, 1 μg/L) was added to each well and continued to culture these cells under the same environment for an additional 1 h. Next, 50 μL of different concentrations of DBDS (0.63, 2.5, 10, and 40 µmol/L) and L-NMMA (positive control, 25 µmol/L) were added to each well and cells were continued to be cultured under the same environment for an additional 24 h, and three repeated wells were set at each concentration. NO production was determined by measuring the accumulation of nitrite in the supernatant of culture medium using the Griess reagent. Inhibition (%) was calculated using the following equation:

$$\mathrm{inhibition}\;(\%)=\;\frac{A\;-\;B}{A\;-\;C}\;\times 100$$

*A*: NO concentration of LPS-treated group; *B*: NO concentration of sample-treated group; *C*: NO concentration of control group.

### ELISA for iNOS, IL-6, TNF-α and COX determination

The logarithmic phase cells were seeded in 24-well plates with 1 × 10^5^ cells/well (volume of the well is 400 μL). Under a humidified atmosphere (5% CO_2_, 37 °C), the cells were cultured for 24 h. Subsequently, 50 μL of LPS (1 μg/L) was added to each well and cells were continued to be cultured under the same environment for an additional 1 h. Next, 50 μL of different concentrations of DBDS (0.63, 2.5, 10, and 40 µmol/L) were added to each well and incubated with cells for an additional 24 h, and three repeated wells were set at each concentration. Then, the supernatant of culture medium was collected and used for measuring iNOS (inducible NO synthase), interleukin6 (IL-6), tumor necrosis factor-α (TNF-α), COX (cyclooxygenases)-1, and COX-2 by ELISA kits. Absorbance was measured in an ELISA reader (Thermo Multiskan GO) at 450 nm. Measurements performed in triplicate. The inhibition on iNOS, IL-6, TNF-α and COX releases was calculated.

### Statistical analyses

All the data are expressed as mean ± s.e.m. All statistical analyses were carried out using the Origin 6.0 software and the GraphPad Prism 6.0 software (GraphPad Prism Software Inc., San Diego, CA, USA). The data obtained were evaluated by One-way ANOVA and unpaired Student’s *t*-test. For GABA-induced dose-response curves, statistical differences were determined using Student’s two tailed *t*-test (Jiang et al. 2011). *p* (adjusted *p* value) < 0.05 indicated a statistically significant difference between two groups.

## Results

### Dolichos trilobus-related compounds relieving pain behaviors on acetic acid-induced writhing model

**Figure 1.**
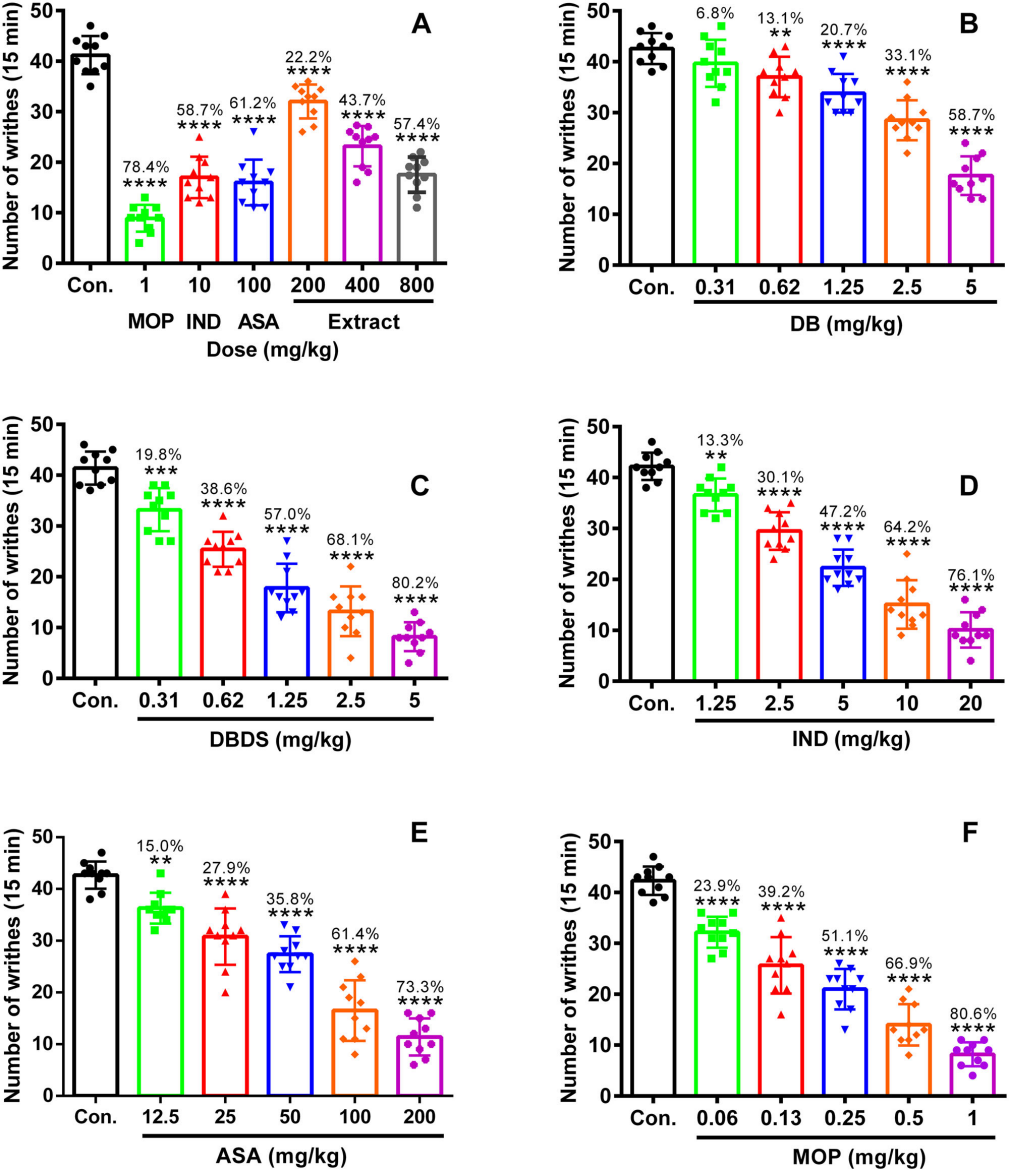
Antinociceptive effect of *D. trilobus*–related compounds on writhing induced by an i.p. injection of 0.6% acetic acid (2 mL) in mice. Mice were orally or intraperitoneally pretreated with different doses of compounds for 30 min. After an i.p. injection of acetic acid, the number of writhing was recorded within 15 min. Data are expressed as mean ± s.e.m. (*n* = 10), **p < 0.01, ***p < 0.001, and ****p < 0.0001 *vs.* control group. One-way ANOVA.

Initially, we performed the acetic acid-induced writhing test. As shown in Figure 1(A), MOP, IND, and ASA, as positive controls, could strikingly reduce the writhing number as comparing to the control group with 78.4, 58.7, and 61.2% inhibition, respectively (F_6, 63_ = 86.12, *p* < 0.0001). The extract of *D. trilobus* exhibited a significant antinociceptive activity at the dose of 200, 400, and 800 mg/kg. The inhibition of 22.2, 43.7, and 57.4% were obtained, respectively (*p* < 0.0001). For the compounds DB and DBDS, when their doses were increased from 0.31 to 5 mg/kg, the number of writhing was progressively decreased (Figure 1(B and C)). DB and DBDS at the dose of 5 mg/kg inhibited the writhing number by 58.7% (F_5, 55_ = 54.58, *p* < 0.0001) and 80.2% (F_5, 55_ = 99.56, *p* < 0.0001), respectively. DB exhibited an ED_50_ (median effective dose) value of 4.68 mg/kg, which is nearly 4-fold weaker than that of DBDS (ED_50_ = 1.1 mg/kg). As a comparison, IND and ASA were tested in the same model as DBDS with an ED_50_ value of 5.91 and 70.09 mg/kg, which are approximately 5 and 64-fold weaker than that of DBDS, respectively. IND at the dose of 20 mg/kg inhibited the writhing number by 76.1% (F_5, 55_ = 117.8, *p* < 0.0001). ASA at the dose of 200 mg/kg inhibited the writhing number by 73.3% (F_5, 55_ = 80.28, *p* < 0.0001). Meanwhile, as positive control, MOP was also tested in the same model with an ED_50_ value of 0.22 mg/kg, which was stronger than that of DBDS. MOP at the dose of 1 mg/kg inhibited the writhing number by 80.6% (F_5, 55_ = 106.9, *p* < 0.0001).

### DBDS relieved thermal pain

Next, we carried out the Eddy’s hot plate test to further evaluate the antinociceptive effect of DB and DBDS. Compared to control group, IND (10 mg/kg) did not exhibited antinociceptive effect with increased paw withdrawal threshold at 0 min (F_11, 108_ = 0.47, *p* > 0.05), 30 min (F_11, 108_ = 9.03, *p* > 0.05), 60 min (F_11, 108_ = 11.52, *p* > 0.05), 90 min (F_11, 108_ = 4.01, *p* > 0.05), and 120 min (F_11, 108_ = 3.62, *p* > 0.05). MOP (1 mg/kg) exhibited outstanding antinociceptive effect, and ASA (100 mg/kg) exhibited particular antinociceptive effect. However, DB and DBDS could only increase withdrawal threshold at the dose of 20 mg/kg (see Table 1, *p* < 0.01).

**Table 1.** Effect of DBDS on hot plate-induced nociception in mice.

Group	Dose (mg/kg)	Pain threshold (s)				
		0 min	30 min	60 min	90 min	120 min
Control	–	11.0 ± 3.1	11.8 ± 3.5	11.5 ± 3.5	10.6 ± 3.3	12.7 ± 3.8
MOP	1	11.3 ± 2.9	23.3 ± 5.9****	23.9 ± 4.9****	19.0 ± 5.3**	18.3 ± 5.2*
IND	10	11.3 ± 2.8	11.7 ± 3.4	12.5 ± 3.0	12.1 ± 2.9	10.9 ± 2.9
ASA	100	11.4 ± 2.8	18.6 ± 5.2*	17.8 ± 3.3**	12.9 ± 4.0	10.6 ± 2.8
DB	2.5	12.0 ± 4.4	12.1 ± 3.7	12.7 ± 4.4	14.2 ± 4.7	12.4 ± 3.4
DB	5	11.4 ± 2.9	12.0 ± 3.4	10.2 ± 2.9	12.6 ± 3.6	10.9 ± 3.7
DB	10	10.4 ± 3.0	14.6 ± 4.9	16.4 ± 6.2	15.6 ± 4.2	12.6 ± 3.7
DB	20	11.8 ± 3.3	18.8 ± 3.6**	19.7 ± 5.3***	18.1 ± 6.3**	13.8 ± 4.2
DBDS	2.5	9.8 ± 2.4	11.1 ± 5.4	11.6 ± 3.8	13.5 ± 4.8	11.3 ± 2.7
DBDS	5	11.3 ± 4.5	12.2 ± 5.7	11.3 ± 2.9	13.5 ± 3.4	11.7 ± 4.2
DBDS	10	10.3 ± 2.8	17.2 ± 3.5	16.2 ± 4.5	14.3 ± 4.3	14.9 ± 6.5
DBDS	20	12.2 ± 3.8	21.8 ± 5.2****	20.9 ± 3.6****	20.3 ± 7.3***	16.9 ± 4.8

Data are expressed as mean ± s.e.m. (*n* = 10), *p < 0.05, **p < 0.01, ***p < 0.001, and ****p < 0.0001 *vs.* control group. One-way ANOVA.

### DBDS relieved pain on formalin test

**Figure 2.**
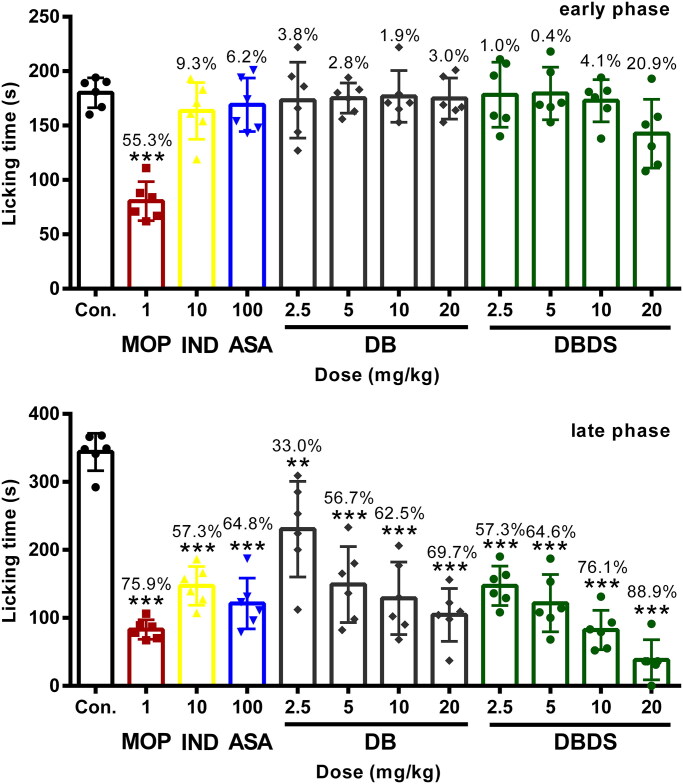
Effect of DBDS on formalin-induced nociception in mice. Mice received vehicle (10 mL/kg, i.g.), MOP (1 mg/kg, i.p.)), IND (10 mg/kg, i.g.), ASA (100 mg/kg, i.g.), DB (2.5, 5, 10, and 20 mg/kg, i.g.), and DBDS (2.5, 5, 10, and 20 mg/kg, i.g.). Data are expressed as mean ± s.e.m. (*n* = 6), *p < 0.05, ***p < 0.001, and ****p < 0.0001 *vs.* control group. One-way ANOVA.

As shown in Figure 2, in the early phase, i.g. administration of DBDS shortened the licking time of mice at the higher dose of 20 mg/kg with 21.8% inhibition, as compared with the control group (F_11, 60_ = 8.21, *p* < 0.05). However, DB exhibited poor antinociceptive effect. In the late phase, DB and DBDS showed a significant inhibition at the doses ranging from 2.5 to 20 mg/kg (F_11, 60_ = 22.74, *p* < 0.001). As expected, IND and ASA mainly acted at the peripheral nervous system (PNS), they could not distinctly shorten the licking time of mice in the first phase, whereas it exhibited remarkable antinociceptive activity in the late phase with 57.3% (*p* < 0.0001) and 64.8% (*p* < 0.0001) inhibition, respectively. As an effective analgesic, MOP showed high antinociceptive activity in both phases with 55.3% (*p* < 0.0001) and 75.9% (*p* < 0.0001) inhibition, respectively.

### Effect of DBDS on LPS-induced inflammatory model

**Figure 3.**
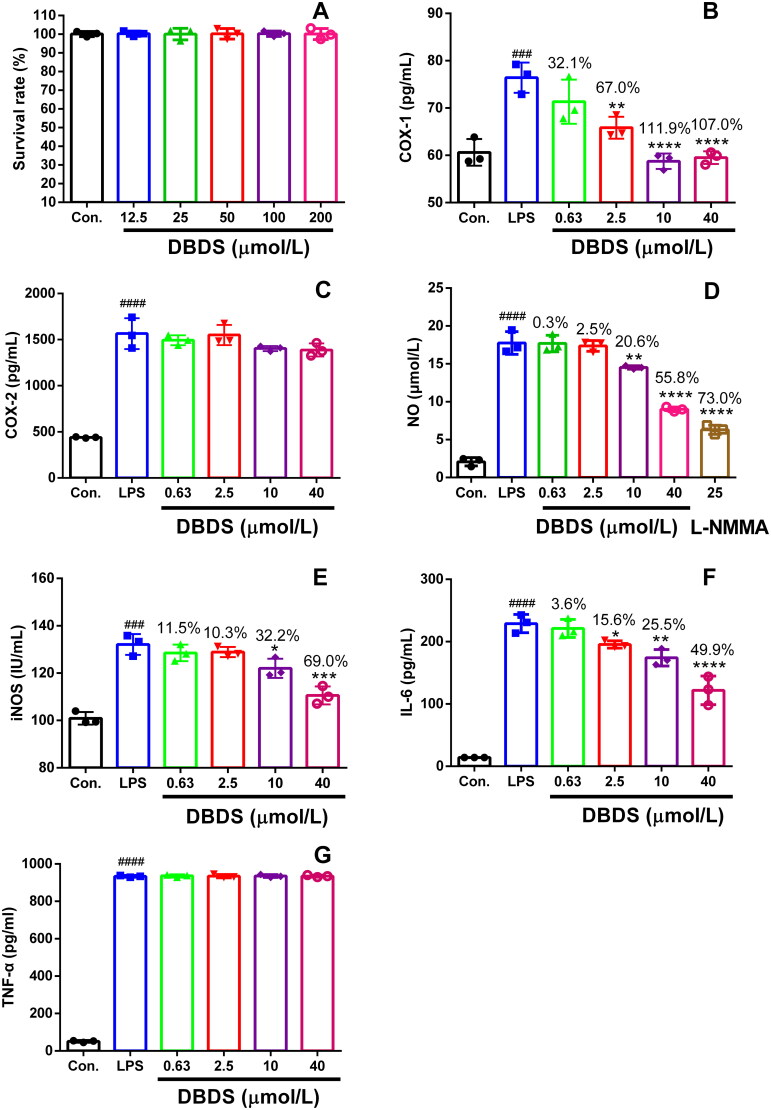
Effect of DBDS on LPS-induced inflammation in RAW264.7 macrophages. Cytotoxicity of DBDS in RAW264.7 macrophages was shown in **A**. Cells were treated with vehicle (control) and DBDS (12.5, 25, 50, 100, and 200 μmol/mL) for 24 h. The survival rate was measured with an MTT assay. Data are expressed as mean ± s.e.m. (*n* = 3). The levels of COX-1, COX-2, NO, iNOS, IL-6, and TNF-α in RAW264.7 macrophages were showed in **B**, **C**, **D**, **E**, **F** and **G**, respectively. The control group was treated with vehicle without LPS and drug. Data are expressed as mean ± s.e.m. (*n* = 3), ^###^*p* < 0.001 and ^####^*p* < 0.0001 *vs.* control group, *p < 0.05, **p < 0.01, and ****p < 0.0001 *vs.* LPS-treated group. One-way ANOVA.

The cytotoxicity of DBDS on RAW264.7 macrophages was firstly tested. The RAW264.7 macrophages were treated with different dose of DBDS. DBDS did not exhibit toxic effects on RAW264.7 macrophages at the concentrations ranging from 12.5 to 200 μmol/L (Figure 3(A), F_5, 12_ = 0.01, *p* > 0.05). Due to the fact that DBDS did not induce significant cytotoxicity in RAW264.7 macrophages, test of DBDS on inflammatory mediators and COX production in LPS-stimulated RAW264.7 macrophages were further performed. As shown in Figure 3(B), COX-1 production was strikingly increased by the treatment of LPS (F_5, 12_ = 18.69, *p* < 0.001). However, it could be effectively reduced by the treatment of DBDS at higher concentrations (2.5, 10, and 40 μmol/L) with 67, 111.9, and 107% inhibition (*p* < 0.01), respectively. After the treatment of DBDS (0.63, 2.5, 10, and 40 μmol/L), no distinct effects were observed on COX-2 production in LPS-stimulated RAW264.7 macrophages (Figure 3(C), F_5, 12_ = 68.74, *p* > 0.05). These results showed that DBDS had effective anti-COX-1 activity with high selectivity.

We also investigated the effects of DBDS on NO, iNOS, IL-6 and TNF-α production in LPS-stimulated RAW264.7 macrophages with an ELISA assay. As shown in Figure 3(D), NO production could be distinctly reduced by DBDS at higher concentrations (10 and 40 μmol/L) with 20.6% (F_6, 14_ = 177, *p* < 0.01) and 55.8% (*p* < 0.0001) inhibition, respectively. Similarly, DBDS could effectively inhibit the production of iNOS at higher concentrations (10 and 40 μmol/L) with 32.2% (F_5, 12_ = 36.51, *p* < 0.05) and 69% (*p* < 0.0001) inhibition, respectively (Figure 3(E)). As shown in Figure 3(F), DBDS clearly reduced IL-6 production at the concentrations of 2.5, 10, and 40 μmol/L with 15.6 (F_5, 12_ = 101.2, *p* < 0.05), 25.5 (*p* < 0.01), and 49.9% (*p* < 0.0001) inhibition, respectively. However, the treatment of DBDS (0.63, 2.5, 10, and 40 μmol/L) could not show significant inhibitory effects on TNF-α production in LPS-stimulated RAW264.7 macrophages (Figure 3(G), F_5, 12_ = 8877, *p* > 0.05).

### Effect of DBDS on the nociception-induced models in the absence and presence of NAL

**Figure 4.**
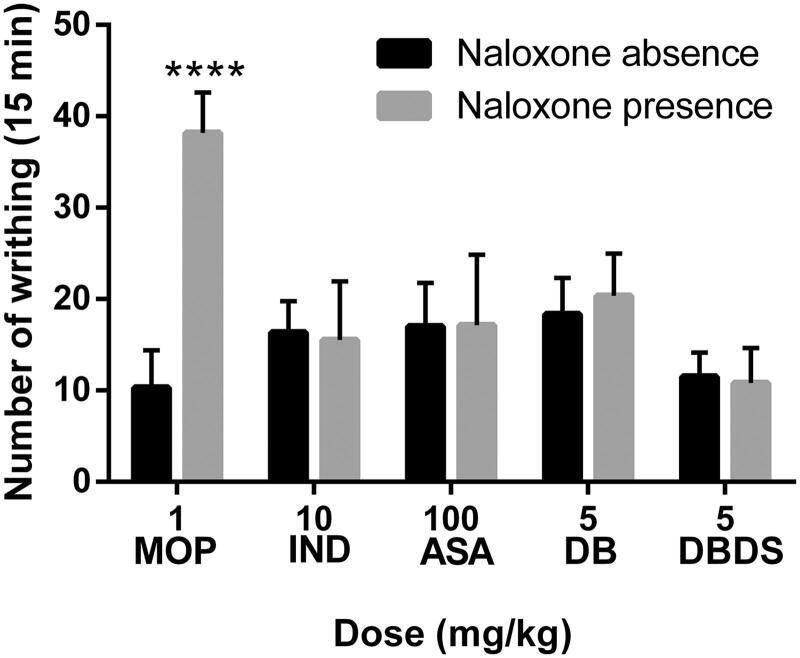
Effects of MOP, IND, ASA, DB, and DBDS on the acetic acid-induced writhing mice in the absence and presence of NAL. Mice were treated with morphine (1 mg/kg, i.p.), IND (10 mg/kg, i.g.), ASA (100 mg/kg, i.g.), DB (5 mg/kg, i.g.), and DBDS (5 mg/kg, i.g.) 30 min before acetic acid injection. The dose of NAL is 5 mg/kg (i.p.). Data are expressed as mean ± s.e.m. (*n* = 6), ****p < 0.0001 *vs.* absence NAL group by Student’s *t*-test.

**Figure 5.**
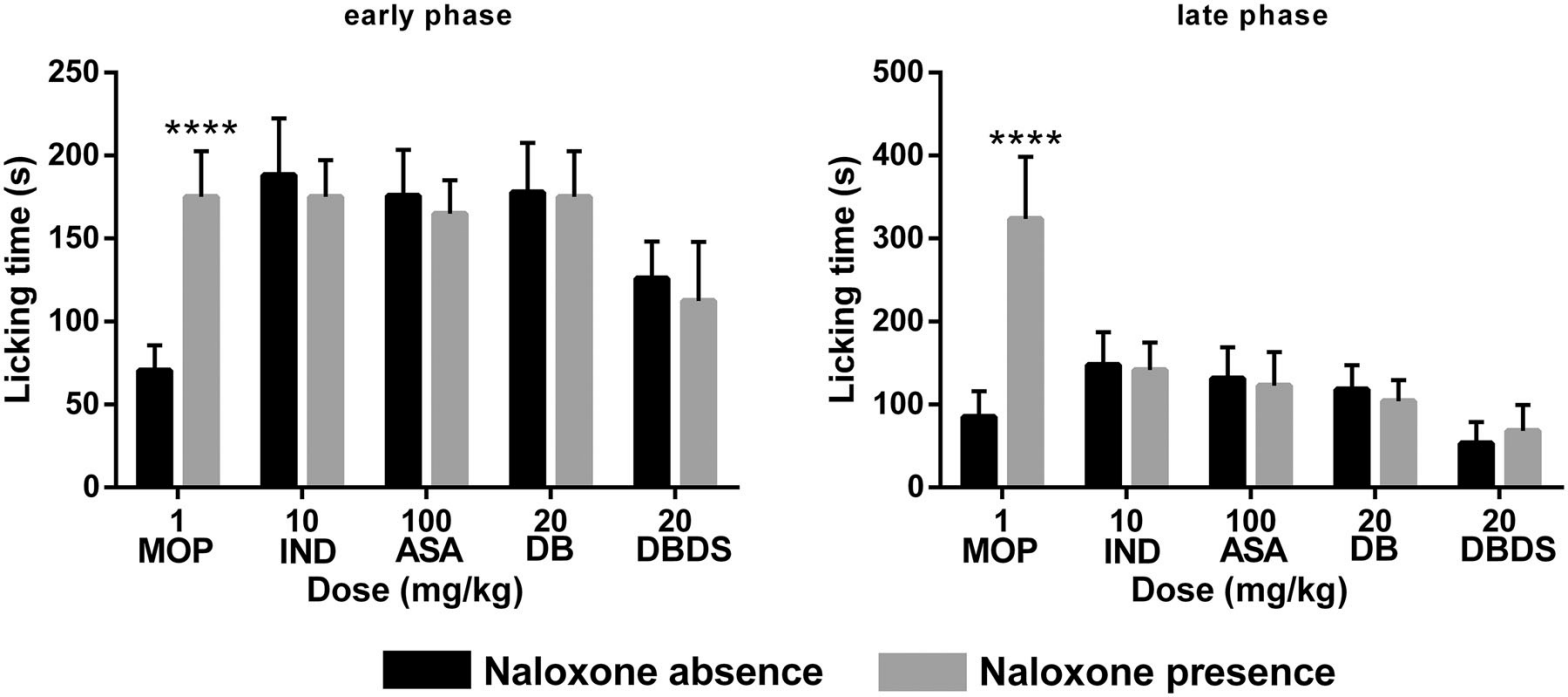
Effects of MOP, IND, ASA, DB, and DBDS on formalin-induced nociception in mice in the absence and presence of NAL. Mice received MOP (1 mg/kg, i.p.), IND (10 mg/kg, i.g.), ASA (100 mg/kg, i.g.), DB (20 mg/kg, i.g.), and DBDS (20 mg/kg, i.g.). The dose of NAL is 5 mg/kg (i.p.). Data are expressed as mean ± s.e.m. (*n* = 6), ****p < 0.0001 *vs.* absence NAL group by Student’s *t*-test.

As shown in Figure 4, MOP could effectively lower the number of writhes of mice in the absence of NAL. However, NAL removed the effect of morphine on the number of writhing (*t* = 11.3, *p* < 0.0001). Only a minor difference was observed when IND (*t* = 0.28), ASA (*t* = 0.05), DB (*t* = 0.8), and DBDS (*t* = 0.35) were tested in the absence and presence of NAL (*p* > 0.05). Similarly, the antinociceptive effect of MOP was removed by NAL in hot plat and formalin tests, and the antinociceptive effect of IND, ASA, DB, and DBDS was not effectively removed by NAL (see Table 2 and Figure 5).

**Table 2.** Effects of MOP, IND, ASA, DB, and DBDS on hot plate-induced nociception in mice in the absence and presence of naloxone.

Group	Dose (mg/kg)	Pain threshold (s)				
		0 min	30 min	60 min	90 min	120 min
MOP	1	11.7 ± 3.7	23.9 ± 5.8	25 ± 4.2	20.7 ± 5.7	17.1 ± 4.4
MOP + NAL	1 + 5	12.0 ± 2.8	12.8 ± 3.6****	11.9 ± 3.3***	13.9 ± 3.6**	10.7 ± 2.5***
IND	10	12.1 ± 2.9	11.7 ± 3.0	11.6 ± 2.2	10.7 ± 2.1	12.0 ± 3.2
IND + NAL	10 + 5	11.4 ± 2.6	11.1 ± 2.5	10.8 ± 4.2	11.4 ± 2.6	11.2 ± 3.2
ASA	100	11.9 ± 2.8	20.3 ± 5.9	19.8 ± 3.2	14.1 ± 4.3	11.6 ± 3.1
ASA + NAL	100 + 5	12.3 ± 4.2	17.7 ± 5.0	17.7 ± 5.1	12.3 ± 4.2	12.1 ± 4.2
DB	20	11.8 ± 3.3	19.6 ± 4.2	21.7 ± 5.9	18.0 ± 5.6	15.2 ± 4.6
DB + NAL	20 + 5	10.6 ± 2.2	18.6 ± 3.7	19.9 ± 5.4	16.5 ± 5.9	11.6 ± 2.8
DBDS	20	12.2 ± 3.8	27.3 ± 6.0	18.2 ± 4.1	20.5 ± 5.7	14.8 ± 2.8
DBDS + NAL	20 + 5	10.9 ± 2.8	20.6 ± 4.3	21.5 ± 5.2	18.8 ± 4.9	13.1 ± 2.0

Data are expressed as mean ± s.e.m. (*n* = 10), **p < 0.01, ***p < 0.001, and ****p < 0.0001 *vs.* absence NAL group by Student’s *t*-test.

**Table 3.** *t* Value basing on Table 2.

Comparisons test	*t* Value				
	0 min	30 min	60 min	90 min	120 min
MOP + NAL *vs.* MOP	0.21	5.16	7.75	3.2	4
IND + NAL *vs.* IND	0.54	0.46	0.57	0.71	0.57
ASA + NAL *vs.* ASA	0.25	1.06	1.11	0.98	0.3
DB + NAL *vs.* DB	0.95	0.55	0.63	0.6	2.07
DBDS + NAL *vs.* DBDS	0.87	2.86	1.53	0.68	1.5

**Table 4.** *t* Value basing on Figure 5.

Comparisons test	*t* Value	
	Early phase	Late phase
MOP + NAL *vs.* MOP	8.16	7.22
IND + NAL *vs.* IND	0.79	0.3
ASA + NAL *vs.* ASA	0.79	0.39
DB + NAL *vs.* DB	0.16	0.9
DBDS + NAL *vs.* DBDS	0.79	0.91

### Activation of DBDS on ion channels

**Figure 6.**
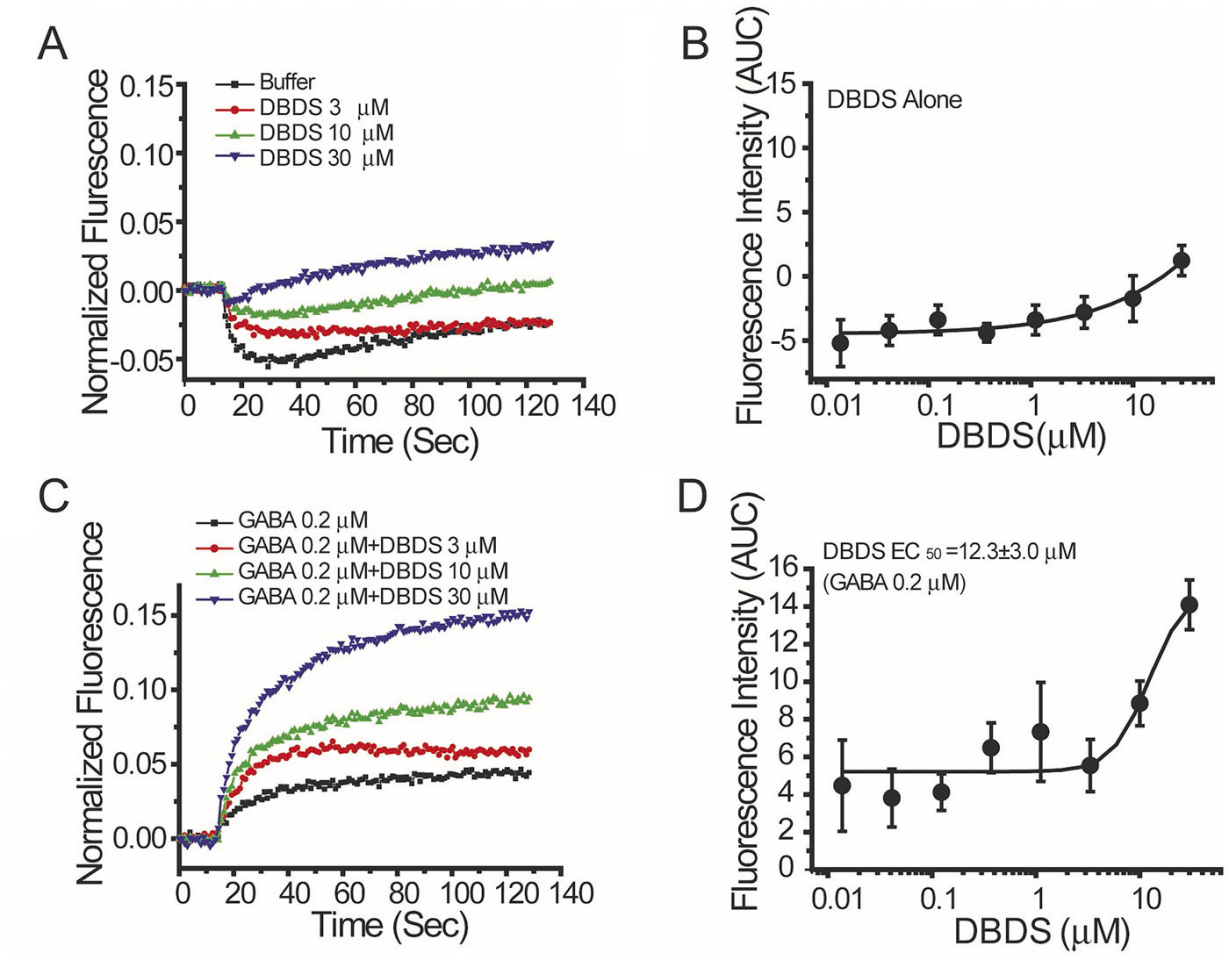
Effect of DBDS on α1β2γ2 GABA_A_ channel. The activity of GABA channel was monitored with FMP-Blue-dye (*n* = 4). (A) The representative traces on GABA_A1_ in the absence and presence of compound DBDS (3, 10, and 30 μM); (B) the dose response curve for DBDS alone; (C) the representative fluorescence traces of compound DBDS (3, 10, and 30 μM) in the presence of GABA 0.2 µM; (D) the dose response curve of the compound DBDS on GABA_A1_ receptor in the presence of GABA 0.2 µM. The dose response curves were fitted with Hill equation by Origin 6.0 software.

As shown in Figure 6(A), when DBDS alone was directly added to the cells, it induced slight activation of the GABA_A1_ channel at higher concentrations (10 µM and 30 µM). However, when an EC_20_ concentration of GABA (0.2 µM) was presented, it was found that DBDS dramatically increased GABA-induced activation of GABA_A1_ channel in a dose-dependent manner with EC_50_ value of 12.3 ± 3.0 µM (Figure 6(D)).

**Figure 7.**
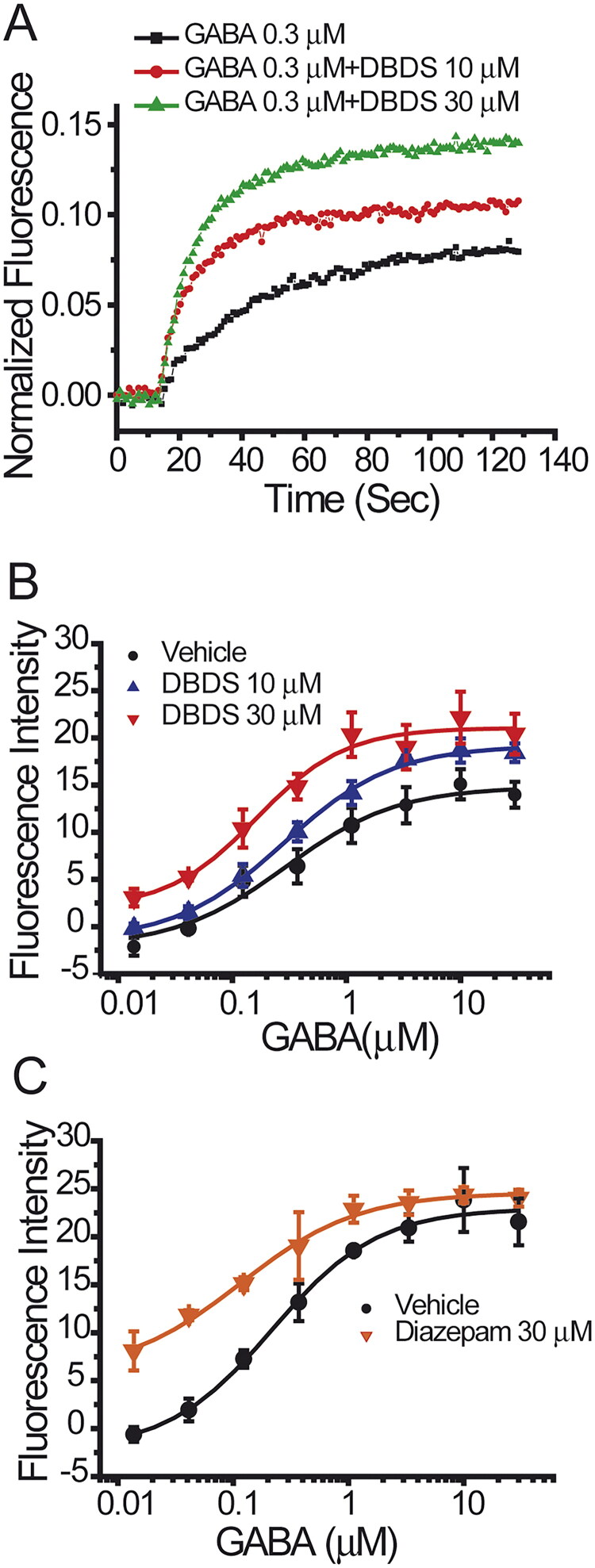
Effect of compound DBDS on dose response curve of GABA in the GABA_A_ α1β2γ2-T-REx™-CHO cells (*n* = 4). (A) The representative traces for GABA from single wells in the absence and presence of compound DBDS (10 and 30 μM); (B) the dose response curve of GABA in the absence and presence of DBDS (10 and 30 μM); (C) the dose response curve of GABA in the absence and presence of diazepam (30 μM).

These data indicated that DBDS is likely a positive allosteric modulator of GABA receptor. Therefore, the effect of DBDS on GABA-induced activation of GABA_A1_ receptor was further studied. In the absence of DBDS, the dose-response curve of GABA on GABA_A1_ receptor was shown in Figure 7(B) (Black filled circles, EC_50_ = 0.29 ± 0.08 µM). As shown in Figure 7, DBDS (10 µM) increased the maximal efficacy of GABA by 30% but did not alter the affinity (EC_50_ = 0.28 ± 0.04 µM) with a similar level to GABA itself (EC_50_ = 0.29 ± 0.08 µM). When the concentration of DBDS was increased to 30 µM, the efficacy of GABA was increased to a higher level by around 150% (*p* < 0.01 *vs.* vehicle control), and the affinity of GABA-induced activation was significantly enhanced by 1.75-fold, with EC_50_ = 0.16 ± 0.05 µM (*p* < 0.01 *vs.* vehicle control), as shown in Figure 7. For GABA_A1_ receptors, diazepam was used as the allosteric control and could shift the EC_50_ value of GABA to 0.12 ± 0.03 µM (Figure 7(C)). These results indicated that DBDS might be allosterically modulating the function of GABA_A1_ receptor.

**Figure 8.**
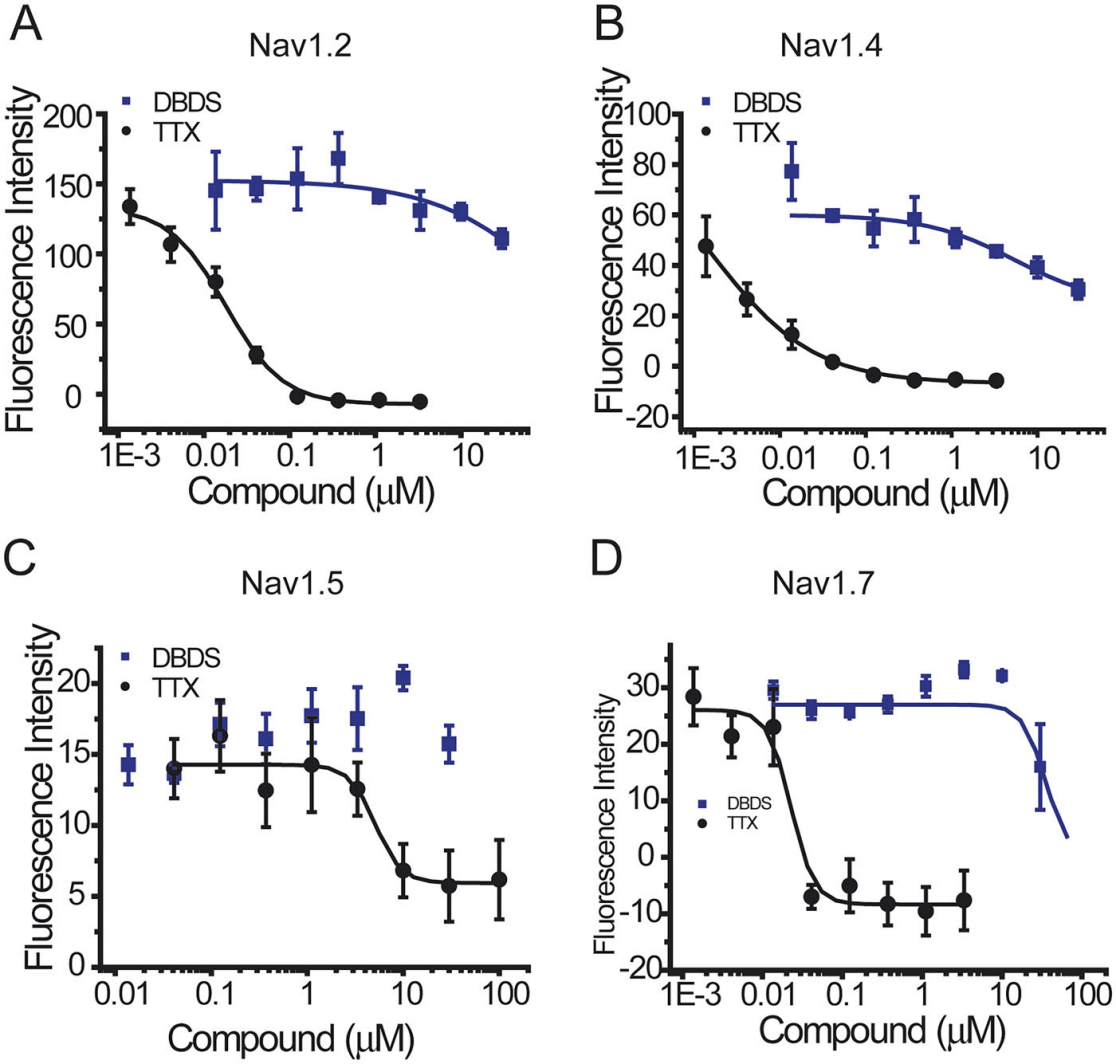
The effect of compound DBDS on sodium channels in the recombinant cells (*n* = 4). The dose-response curves for Nav1.2 (**A**), Nav1.4 (**B**), Nav1.5 (**C**), and Nav1.7 (**D**). FMP-Blue-Dye was used. The following cell lines were used, Nav1.2-CHO-FlpIn cells, Nav1.4-CHO-K1 cells, Nav1.5-HEK293 cells and Nav1.7-HEK-FlpIn cells.

**Figure 9.**
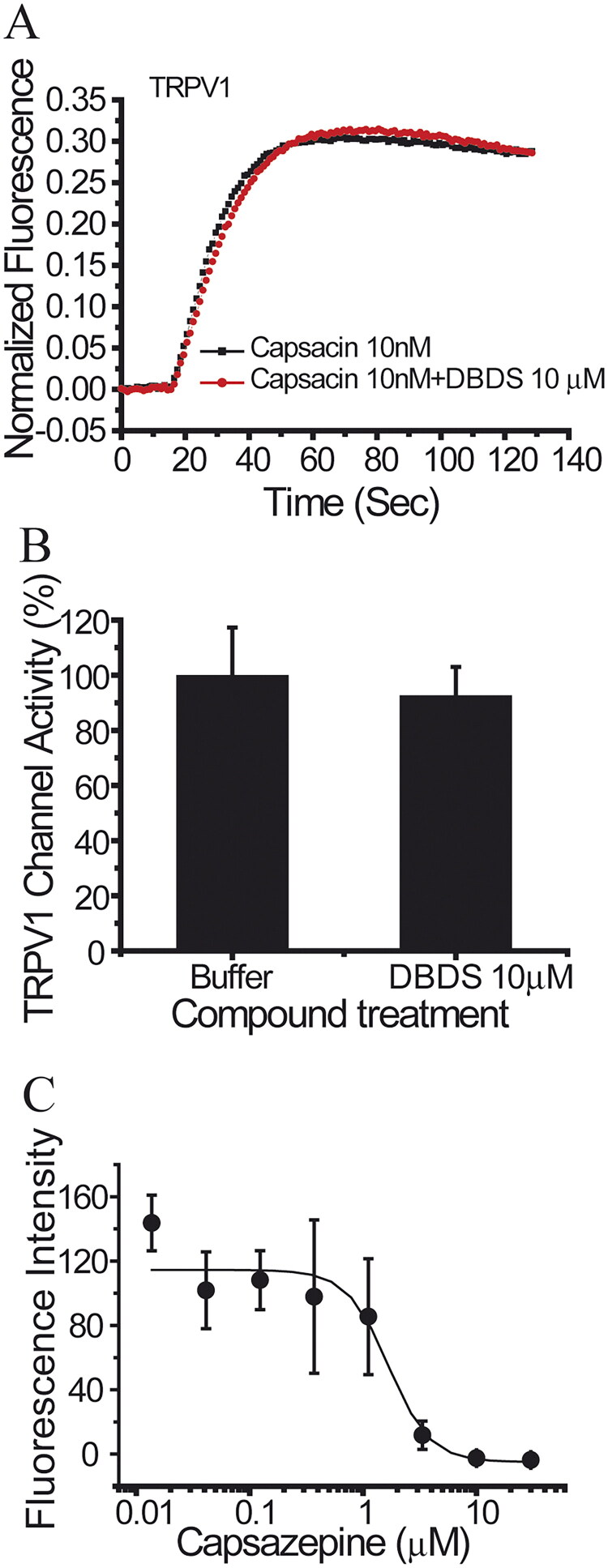
The effect of compound DBDS on TRPV1 channel expressed in HEK293 cells (*n* = 4). (A) Representative traces in the absence and presence of DBDS (10 μM) on the capsaicin-induced TRPV1 signal; (B) bar graph for the activity change of DBDS (10 μM) on TRPV1; (C) activity change of capsazepine on TRPV1.

Similar to GABA channel, FMP assay was used for the functional test of sodium channels. As shown in Figure 8, DBDS has weaker effect on peripheral sodium channel Nav1.7 (IC_50_ = 32.62 ± 4.90 µM), central nervous system (CNS) subtype Nav1.2 (IC_50_ = 140.67 µM), and stronger inhibition on skeletal muscle subtype Nav1.4 (IC_50_ = 3.29 ± 2.12 µM). Fortunately, DBDS has no effect on Nav1.5 subtype of cardiac sodium channel (Figure 8(C)), a potential target of cardiac safety. Tetrodotoxin (TTX) was used as the positive control, and the half inhibitory concentrations are 0.016 ± 0.015 µM for Nav1.2, 0.005 ± 0.001 µM for Nav1.4, 3.4 ± 0.24 µM for Nav1.5, and 0.02 ± 0.003 µM for Nav1.7channels. In the meanwhile, we also tested the effect of DBDS on another pain target – TRPV1 channel. As shown in Figure 9, TRPV1 activator capsaicin induced a significant activation of TRPV1 channel, but DBDS (10 µM) did not alter the activity of TRPV1. In addition, we also tested the direct effect of DBDS and found that DBDS alone could not induce any change of TRPV1 activity. For TRPV1 channel, capsazepine was used as an inhibitor control (IC_50_ = 7.3 ± 0.06 µM).

## Discussion

To identify the analgesic constituents from *D. trilobus*, we prepared the ethanol extract and carried out the isolation of extract according to previously reported procedures by our group (Luo et al. 2021). We obtained a series of compounds from the extract, including doliroside A and B, among which DB had the highest percentage of content. It also naturally existed in a salt form in *D. trilobus* (Feng et al. 1985). Thereafter, its disodium salt was generated through the combination of compound DB and sodium hydroxide in methanol solution. Subsequently, the antinociceptive effect of DB, as the main constituent of *D. trilobus*, and DBDS was evaluated on the nociception-induced models.

The acetic acid-induced writhing model has been widely utilized as a standard assay to explore compounds with peripheral antinociceptive activity from natural sources (Ayumi et al. 2020). It was widely employed to evaluate the antinociceptive activity of triterpenoid saponins (Wang et al. 2006; Speroni et al. 2007; Yang et al. 2008; Chen et al. 2018). The injection of acetic acid stimulates peripheral tissue damage, which further promotes the synthesis of eicosanoids – an important mediator in pain and inflammation, and then results in the release of various inflammatory mediators and prostaglandins – a main substance sensitizing the nociceptive neuron and inducing hyperalgesia – which plays a key role in the writhing behaviors in this test (Berkenkopf and Weichman 1988). Therefore, we used the acetic acid-induced writhing model to evaluate the antinociceptive activity of *D. trilobus*-related compounds. Among them, DBDS showed the highest antinociceptive activity. The observed high antinociceptive activity of DBDS in the acetic acid-induced writhing test suggested that it could produce peripheral antinociception might related to the mechanism like peripherally acting drugs such as NSAIDs.

Stimulation of hot plate generates a noninflammatory and acute nociceptive response appeared as paw licking and jumping behaviors occurring at the supraspinal level (Le Bars et al. 2001). Therefore, a drug or substance possesses centrally mediated activity as opioids if its paw withdrawal threshold increased (Abdul Rahim et al. 2016). The antinociceptive activity of triterpenoid saponins was widely evaluated by this animal model (Wang et al. 2006; Speroni et al. 2007; Yang et al. 2008; Arrau et al. 2011; Chen et al. 2018). To demonstrate the antinociceptive activity of DBDS is effective in CNS, we tested its effect through the Eddy’s hot plate test. Testing results demonstrated that DBDS exhibited antinociceptive activity at higher dose possibly through the CNS.

The formalin test has been commonly used to verify the participation of peripheral and central analgesic activities of compounds. It is a frequently-used animal model to evaluate the antinociceptive activity of triterpenoid saponins (Speroni et al. 2007; Chen et al. 2018; Sun et al. 2019). Therefore, the effect of DBDS on formalin test was examined. An intraplantar injection of formalin could immediately activate the nociceptors and produce abnormal repetitive behaviors, such as licking and/or biting of the injected paw, lasting about 5 min (Shibata et al. 1989). This is the early phase, in which the noninflammatory/neurogenic pain will occur. After 15 min of formalin administration, bradykinin, prostaglandins, and histamine will be released, causing inflammatory pain. Then the late phase of the formalin test started and lasted for about 15 min. It is well-known that the centrally acting drugs can relieve pain in the early phase of the test, and the drugs acting both at PNS and CNS can suppress pain in the late phase (Sulaiman et al. 2010). DBDS exhibited significant antinociceptive activity both in the early and late phase in formalin test, indicated that it is a potential analgesic acting on both PNS and CNS.

Due to the potent analgesic activity of DBDS in the acetic acid-induced nociceptive model, we planned to have an insight into its antinociceptive mechanism. Injection of acetic acid, which caused inflammatory pain, could promote the synthesis of arachidonic acid. Arachidonic acid catalyzed by COX can form various prostaglandins (PG). As one of them, PGE_2_ can induce hyperalgesia. Hence, reduction of COX is an effective pathway to relieve inflammatory pain. Therefore, we decided to test its activity on COX production in LPS-stimulated RAW264.7 macrophages. Testing results showed that DBDS had effective anti-COX-1 activity with high selectivity. Though COX-2-selective agents provide good anti-inflammatory and analgesic actions, it would increase the risk of cardiovascular diseases when these are taken chronically. In contrast, COX-1-selective agents could decrease the risk of cardiovascular diseases by reducing the production of thromboxane A2. Therefore, DBDS would be a potential candidate of antinociceptive agent used clinically for patients with a history of myocardial or cerebral infarction.

In the process of inflammatory pain, inflammatory mediators, such as NO and cytokines, play important roles between the disease process and pain. NO can directly damage the function of normal cells through binding with other superoxide radicals, which is an important inflammatory mediator during the inflammatory process (Moncada et al. 1991). Over production of NO in the inflammatory process is associated with pain behaviors. Meanwhile, study reports show that IL-6 and TNF-α play a key role in the progress of inflammatory pain (Wei et al. 2015). Therefore, we investigated the effects of DBDS on NO, iNOS, IL-6, and TNF-α production in LPS-stimulated RAW264.7 macrophages. Results showed that DBDS could also distinctly reduce the productions of NO, iNOS, and IL-6, which could be very important for relieving inflammatory pain.

It is well-known that many different nociceptive mechanisms involved in the acetic acid-induced nociceptive model, such as the sympathetic system (biogenic amines release), cyclooxygenases and their metabolites (Duarte et al. 1988) and opioid mechanisms (Collier et al. 1968). To explore whether the antinociceptive effect of DBDS is related to opioid receptors, we carried out the acetic acid-induced writhing test, hot plate test, and formalin test in the absence and presence of NAL – an antagonist of opioid receptor. We found that the endogenous opioid peptidergic system was not involved in the antinociceptive activity of DBDS.

Ion channels also play an important role in the occurrence of pain, DBDS exhibited antinociceptive activity which was possibly related to ion channels, whereupon we decided to investigate its possible antinociceptive mechanism on several ion channels, such as GABA_A1_, Nav1.7 and TRPV1 channels. GABA_A_ receptors are widely expressed in brain and spinal cord pain circuits as the principal mediator of inhibitory neurotransmission. Several studies demonstrated that modulation of GABA_A_ receptors showed great potentials for pain alleviation (Knabl et al. 2009; Bonin et al. 2011; Munro et al. 2013; Klinger et al. 2015). GABA_A1_ (α1β2γ2) is the most abundant isoform among GABA receptors. Therefore, the effect of DBDS on GABA_A1_ receptor was tested by using FMP-Blue-Dye method. For the FMP assay in the recombinant GABA_A1_ receptors expressed in T-REx™-CHO cells, as reported (Joesch et al. 2008), it was observed that exposure of agonist GABA resulted in depolarization of cells. In immature neurons, GABA_A_ receptor agonists also depolarized the membrane potential, in contrast to hyperpolarizing in mature cells. These might be caused by intracellular Cl^−^ accumulation due to the absence of Na-K-2Cl cotransporter (Plotkin et al. 1997). Therefore, GABA-induced depolarization in the recombinant cells might be the same as the immature neurons. Through ion channel assay, we found that compound DBDS might be allosterically modulating the function of GABA_A1_ receptor.

Sodium channels are important targets of pain. Genetic studies of patients and animals indicated that neuronal sodium channel Nav1.7 also plays key role in pain sensitization (Cox et al. 2006; Drenth and Waxman 2007). In the present study, we also tested the effects of DBDS on subtype Nav1.7 and its family members. However, its activity on subtype Nav1.7 and its family members were weak except Nav1.4. TRPV1 channel as a previously popular studied pain target represents a promising therapeutic target (Brandt et al. 2012). We also tested the effect of DBDS on TRPV1 channel. However, it did not alter the activity of TRPV1.

## Conclusions

We demonstrated that compound DBDS exhibited potent antinociceptive activity acting on both the PNS and CNS. We found that DBDS had highly selected anti-COX-1 activity. Meanwhile, it plays an important role in relieving pain by reducing the production of NO, iNOS, and IL-6. Through the investigation of ion channels, DBDS could also positively modulate the function of GABA_A1_ receptor. As a potential candidate drug for the treatment of pain, compound DBDS has displayed a great potential for pain alleviation, while the metabolism and toxicity of the compound need to be further explored.

## Supplementary Material

Supplemental Material
