# Supplementary material for: Antinociceptive activity of doliroside B
Source: Pharm Biol. 2023 Jan 10;61(1):201–12. doi: 10.1080/13880209.2022.2163407 (PMC9848282; doi:10.1080/13880209.2022.2163407)
Supplement: Supplemental Material [file IPHB_A_2163407_SM5884.docx]

**Supplementary material**

**Figure S1.** ^1^H NMR spectrum of DB (DMSO-d_6_, 400 MHz).

**Figure S2.** ^13^C NMR spectrum of DB (DMSO-d_6_, 100 MHz).

**Figure S3.** ^1^H NMR spectrum of DBDS (D_2_O, 400 MHz).

**Figure S4.** ^13^C NMR spectrum of DBDS (D_2_O, 100 MHz).
